# Assessment of 105 Patients with Angiotensin Converting Enzyme-Inhibitor Induced Angioedema

**DOI:** 10.1155/2017/1476402

**Published:** 2017-02-14

**Authors:** Eva Rye Rasmussen, Christian von Buchwald, Mia Wadelius, Sumangali Chandra Prasad, Shailajah Kamaleswaran, Kawa Khaled Ajgeiy, Georg Authried, Kristine Appel U. Pallesen, Anette Bygum

**Affiliations:** ^1^Department of Otorhinolaryngology, Head & Neck Surgery and Audiology, Rigshospitalet, University of Copenhagen, København, Denmark; ^2^Department of Medical Sciences, Clinical Pharmacology and Science for Life Laboratory, Uppsala University, Uppsala, Sweden; ^3^Department of Dermatology and Allergy Centre, Odense University Hospital, Odense, Denmark

## Abstract

*Objective.* To asses a cohort of 105 consecutive patients with angiotensin converting enzyme-inhibitor induced angioedema with regard to demographics, risk factors, family history of angioedema, hospitalization, airway management, outcome, and use of diagnostic codes used for the condition.* Study Design.* Cohort study.* Methods*. This was a retrospective cohort study of 105 patients with angiotensin converting enzyme-inhibitor induced angioedema in the period 1995–2014.* Results*. The cohort consisted of 67 females and 38 males (F : M ratio 1.8), with a mean age of 63 [range 26–86] years. Female gender was associated with a significantly higher risk of angiotensin converting enzyme-inhibitor induced angioedema. 6.7% had a positive family history of angioedema. Diabetes seemed to be a protective factor with regard to angioedema. 95% experienced angioedema of the head and neck. 4.7% needed intubation or tracheostomy. 74 admissions took place during the study period with a total of 143 days spent in the hospital. The diagnosis codes most often used for this condition were “DT783 Quincke's oedema” and “DT78.4 Allergy unspecified”. Complement C1 inhibitor was normal in all tested patients.* Conclusion*. Female gender predisposes to angiotensin converting enzyme-inhibitor induced angioedema, whereas diabetes seems to be a protective factor.

## 1. Introduction

Angioedema is a transient nonpitting swelling of the dermis and subcutis or submucosa which occurs in a variety of diseases. Asphyxiation is a risk when angioedema localizes in the oral cavity, pharynx, or larynx [1]. When the physician encounters patients with severe acute and/or recurrent angioedema, both treatment and assessment might be a challenge [2]. Angioedema can occur as an adverse effect to several medications, but one drug class is overrepresented, namely, angiotensin converting enzyme-inhibitors (ACEi) [1, 3]. Over the past twenty years, it has become evident that ACEi induced angioedema is frequently misdiagnosed as allergic reactions resulting in the use of noneffective antiallergic medications and failure to withdraw the offending drug [2, 4]. In a few cases, a genetic predisposition to ACEi induced angioedema has been found [5–7]. Seemingly, ACEi induced angioedema is related to polygenetic and demographic as well as environmental factors such as age, race, gender, comorbidity, and smoking, but most available studies are describing heterogeneous and relatively small sample sizes [1, 8]. Also standardized clinical assessments of patients regarding other causes of angioedema are often lacking.

This retrospective study of the clinical characteristics of patients suffering from ACEi induced angioedema was carried out at a Danish Department of Dermatology and Allergy Centre. Patients referred to the department had a history of severe and/or recurrent angioedema of unknown cause and were thoroughly assessed using a systematic angioedema guideline. The main objectives of the study were to describe a large presumed Caucasian cohort with ACEi induced angioedema. Furthermore, previously identified risk factors would be assessed to see whether we could validate the findings. Also, the study served as a quality study for our department, which holds the regional service for angioedema assessment.

## 2. Materials and Methods

A retrospective study of patients diagnosed with ACEi induced angioedema in the period 1995–2014 was performed at a single department of dermatology and allergology, Odense University Hospital, which serves a population of 1.2 million people. Patient data was entered into a database in order to ensure correct and reliable data collection. Stata® version 14 (StataCorp. 2015,* Stata Statistical Software: Release 14*. College Station, TX: StataCorp LP.) was used as database system and for analysis. For statistical analysis, differences of proportion test together with multivariate logistic regression analyses were used. *p* values ≤ 0.05 were considered statistically significant. Relative risk calculation was based on the study population and data (ACEi treated males and females) from the Danish registry on sale of pharmaceuticals 1996–2014 [9]. As there was no control group, statistics was performed comparing groups of patients with each other (i.e., males and females, smokers and nonsmokers, and patients with and without hypertension).

### 2.1. Ethics

This study was approved by the Danish Data Protection Agency (jr. number 14/35206) and the Danish National Board of Health (jr. number 3-3013-805/1/) as appropriate.

## 3. Results

**Figure 1:**
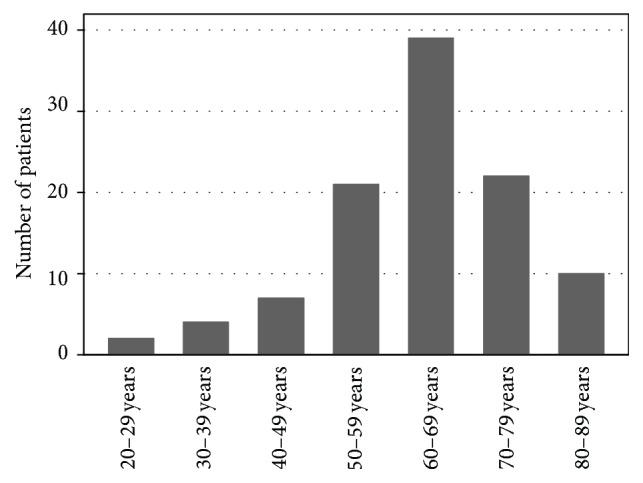
Age distribution of the cohort.

Of 734 consecutive patients assessed due to angioedema, 105 patients were found to have ACEi induced angioedema and were included in the study (Table 1). There was a predominance of females and the mean age was 63 years [range 26–86 years]. The age distribution is shown in Figure 1. The female overrepresentation reached statistical significance (*p* = 0.006; 95% confidence interval (CI) 0.09–0.47). Using MedStat.dk, which is a tool to extract data on prescribed pharmaceuticals in Denmark, we found that 23% more males than females receive an ACEi in the study period [9]. The relative risk of angioedema due to ACEi in females versus males was found to be 1.4 [9].

Patients were most often referred by their general practitioner; 9.5% of them were referred twice due to recurrent angioedema episodes after withdrawal of ACEi. Most patients had experienced more than one angioedema episode prior to referral (Table 1). Relatively few patients needed airway management (Table 2).

More than half the patients had been admitted to hospital due to angioedema at least once with a total of 74 admissions and 143 patient-days spent in hospital (Table 3). Furthermore, 48 of 100 patients had been assessed at an Emergency Department due to angioedema, while the history of Emergency Department visits was unknown for five patients (Table 3).

A variety of diagnostic codes (International Classification of Diseases version 10) were used when admitting angioedema patients with “DT 78.3 Quincke's oedema” and “DT 78.4 Allergy without specification” used in 75–90% of cases in both the Emergency Department and other in-patient departments (Table 3).

As a group, patients with ACEi induced angioedema had many concomitant diseases, predominantly associated with smoking, alcohol use, obesity, and allergic diseases. Using difference of proportion tests, the incidence of hypertension was not surprisingly overrepresented (*p* < 0.0001; 95% CI 0.66–1.04). Diabetes was shown to be a significant protective factor (*p* < 0.005; 95% CI 0.37–0.75) (Table 4). The risk of angioedema was not found to correlate with smoking (Table 1).

In a minor fraction of patients, C-reactive protein or tryptase was elevated (Table 5). None of the patients with recurrent angioedema after ACEi withdrawal had a positive histamine-release (HR) test or elevated tryptase, but three had concomitant rash. All tested patients had normal complement C1 inhibitor levels and function.

Obvious parameters associated with admission to a hospital were intubation and tracheostomy. In multivariate logistic regression analysis, angioedema located in the head and neck region was nonsignificantly associated with admission (odds ratio (OR) 5.9 (*p* = 0.15, 95% CI 0.53–98.49)), while angioedema in peripheral sites was significantly associated with not being admitted (OR 0.15 (*p* = 0.05 95% CI, 0.01–0.95) (Table 6)).

## 4. Discussion

Our study confirmed female gender as a risk factor of ACEi induced angioedema (*p* = 0.006), which previously has been disputed [10]. A possible bias in our study could be more women than men being treated with an ACEi. To assess this, we used MedStat.dk, which is a tool to extract data on prescribed pharmaceuticals in Denmark [9]. In fact, the prescription rate of ACEi for men was 23% higher than that for women, even though there are more women than men in this age group (typically > 45 years of age). This further consolidates female gender as a risk factor. In hereditary angioedema, symptoms are often worse in women, presumably due to an interaction with estrogen, but this has never been studied in ACEi induced angioedema [11].

Smoking has been identified as a risk factor in earlier studies, but no association was found in this study [12, 13]. Diabetes seems to be a protective factor, which was confirmed by our study (21% with versus 79% without diabetes, *p* < 0.005) [14]. The negative correlation between diabetes and ACEi angioedema has not been fully understood. However, it seems that poor blood glucose control and high levels of HbA1c might increase the level of Dipeptidyl-Peptidase IV [15]. This enzyme is one of the main metabolizers of the vasoactive molecules bradykinin and substance P, which are suspected to be the primary mediators for ACEi angioedema. In addition, low levels of Dipeptidyl-Peptidase IV have previously been correlated with ACEi angioedema [8].

Allergic rhinitis, asthma, and atopic dermatitis were not significantly associated with ACEi angioedema in this study. However, a few patients had positive HR test (2.9%) and/or elevated tryptase (5.7%). Those patients might have an underlying mast cell driven condition, which raised their a priori risk of angioedema during ACEi treatment. It has been proposed that an initial histamine-release reaction can set off bradykinin-mediated angioedema attacks, although this needs clarification [16, 17].

6.7% had a positive family history of angioedema, but none of them had complement C1 inhibitor deficiency. Angioedema is relatively prevalent in the general population, and some families might have a genetic predisposition that is not yet understood [18, 19]. As expected, all tested patients had normal levels of complement C1 inhibitor.

Every fifth patient in our cohort had a description of rash/urticaria in their medical records. However, we suspect that both patients and physicians have trouble distinguishing between urticaria and angioedema, as some describe angioedema as “giant hives.” Usually, ACEi angioedema is not associated with urticaria.

It has been proposed that a known idiopathic angioedema could increase the risk of attacks, when treated with ACEi, but as no data were available on idiopathic angioedema prior to ACEi treatment, we were not able to study this further [20]. African descent is another known risk factor both for angioedema due to ACEi and for the need for airway management, signifying a severe angioedema [1, 21]. Our cohort did not include any patients of African descent, so we were not able to verify this previous finding.

The number of patients in need of acute airway management was a little higher than in a previous American study by Tai et al.: intubation 3.8% versus 3.3% and tracheostomy 0.9% versus 0.3% [1]. However, an earlier study from 2007 by Grant et al. displays a larger proportion of intubations, namely, 10% [22]. The demographics in the United States of America and Denmark differ with regard to race, as more people in the US have African ancestry; this might have significance when comparing studies [22]. In the literature, an algorithm has been proposed for assessment and treatment of angioedema in the acute phase [23]. This is an important notion, since the hypopharynx and larynx are not visualized by a routine inspection of the oral cavity. In patients not assessed by an otorhinolaryngologist, close attention should be paid to clinical clues to a potential hypopharynx or larynx swelling: inability to swallow foods/liquids, changed voice, hoarseness, drooling, patient stating to feel a “lump in the throat,” and stridor. Angioedema due to an ACEi tends to have a more severe clinical presentation than most other forms of angioedema, and deaths due to airway obstruction have been described [24].

The recurrence of angioedema in patients on continued ACEi treatment is 187 per 1000 patient-years, but with a rather long latency—on average 11 months to the next attack [25]. All patients in the present study had their ACEi discontinued after assessment at our department. During the follow-up period of 700 patient-months, 10 of 105 (9.5%) patients experienced recurrent angioedema after the withdrawal of ACEi. A national registry based study found a similar recurrence rate of nine percent, while two retrospective clinical studies found higher recurrence rates [25–27]. A limitation of our study is that patients with mild or infrequent angioedema episodes might not have been rereferred to our department and thus not registered with recurrence. Mahmoudpour et al. found that 32–53% of patients with a first-time angioedema attack discontinued ACEi in 2007–2013 (increasing over the time period) [26]. Medical guidelines advise that ACEi should be replaced with an angiotensin 2-receptor blocker (ARB) if adverse drugs reactions occur [28]. In a recent study of ACEi adverse reactions, 43% of angioedema patients were switched to an ARB, 14% switched to “other hypertensive drugs,” and 14% stopped without replacement therapy; the remainder stayed on ACEi [29]. Angioedema recurrence rates were not statistically different between patients that switched to various alternative hypertensive drugs (ARB 34%, calcium antagonists 28% and “other” 29%, *t-*test and Mann–Whitney* U* test *p* > 0.05) [30]. One hypothesis is that ACEi treatment can be the trigger of a latent predisposition to (idiopathic) angioedema. However, genetic factors are also involved in ACEi angioedema, and in one patient multiple enzyme deficiencies were seen [5, 31].

The efficacy of antihistamines (62.5%) and/or corticosteroids (63.3%) reported by patients and physicians are most likely explained by a high spontaneous remission rate of ACEi angioedema, as the bradykinin pathway is not affected by classical antiallergic medication. It is noteworthy that we find the reported efficacy of antiallergic treatments is the same as for the newer drug icatibant, which is a selective bradykinin-2 antagonist. The low number of treated patients does not allow for any conclusions, but a previous study would suggest icatibant to be more effective than antiallergic treatment [32]. One of the important focus areas of medical science—personalized medicine—is aiming to find genetic and clinical factors that predict patients at risk of ACEi induced angioedema, and to these patients another drug should be prescribed. Until such tests and prediction models are available, physicians and patients rely on a thorough assessment of patients with angioedema in order to distinguish between ACEi induced and other types of angioedema.

## 5. Conclusion

In conclusion, this study validates older age and female sex as risk factors of ACEi angioedema, while smoking habits and allergies were not associated. An important finding was that patients with diabetes seem to be protected against ACEi angioedema occurrence; this fact should be explored in upcoming studies. Patients with angioedema of the head and neck were admitted to hospital to a high extent due to the risk of airway obstruction, whereas patients with peripheral angioedema were less likely to be admitted.

## Figures and Tables

**Table 1:** Basic data.

Parameter	
Patients, *n*	105
Male : female	38 : 67
Caucasians	104 (99%)
Age, mean, *years*	63 [range 26–86] (SD 12.42)
Referred > 1 due to recurrent angioedema, *n (%)*	10 (9.5%)
Referring medical specialty	
(i) General practitioner	47
(ii) Internal medicine	28
(iii) Emergency department	11
(iv) Otorhinolaryngology	10
(v) Referred from 2 specialties	6
(vi) Dermatology	1
(vii) Allergy centre	1
(viii) Unknown	1
Follow-up, *patient-months (mean)*	700 (6.7)
History of drug rash	
No	72.3%
Yes	27.6%
History of allergic disease	
No	76.1%
Yes	22.0%
Unknown	1.9%
Smoking	
No	38.0%
Yes	24.8%
Unknown	37.1%
Family history of angioedema	
No	75.2%
Yes	6.7%
Unknown	18.0%
Number of angioedema episodes prior to referral	
1	20.0%
2	7.6%
3–5	17.1%
6–10	9.5%
11–20	4.8%
21–50	1.9%
>50	8.6%
Unknown	30.5%

**Table 2:** Descriptive angioedema incident data. Some patients had experienced angioedema in more than one location; thus, the numbers does not add up to 100.

Parameter		
Localization of angioedema, *n*, *(%)*		
(i) Head and neck		100 *(95.2%)*
(ii) Peripheral		14 *(13.3%)*
(iii) Abdominal		3 *(2.9%)*
Concomitant rash, *n*, (%)		19 *(18%)*
Treatment of acute attacks, *n*, *(%)*		
Antihistamines	96 *(91.4%)*	Efficacy reported by 62.5%
Corticosteroids	79 *(75.2%)*	Efficacy reported by 63.3%
Adrenaline	20 *(19.0%)*	Efficacy reported by 15.0%
Icatibant	3 *(2.9%)*	Efficacy reported by 66.7%
Tranexamic acid	1 *(<1%)*	Efficacy reported by 0%
Beta-2 agonists	1 *(<1%) *	Unknown effect
Montelukast	2 *(1.9%)*	Unknown effect
Azathioprine	1 *(<1%)*	Unknown effect
Airway management		
Intubation	4 patients	3.8%
Tracheostomy	1 patient	0.9%

**Table 3:** Data regarding hospital admissions and Emergency Department visits. Data was unknown regarding admissions in two patients. Three patients were admitted, but the number of admissions was unknown. Thus, they count as one admission each, even though some might have been admitted more than once; therefore, the number of hospitalizations is an approximated minimum. In five patients, the duration of admission was unknown. Diagnostic codes are from the International Classification of Diseases, Tenth Edition *(ICD-10)*.

Hospitalization data	
Patients assessed at Emergency Department	48
Patients admitted to a hospital	55
Number of admissions, total	74
1	39 patients
2	7 patients
3	2 patients
4	4 patients
Unknown number	3 patients
Days of admission, total	143
Days of admission, mean, [range]	2.9 [1–35]
Department of initial admission	
(i) Internal Medicine	40
(ii) Otorhinolaryngology	19
(iii) Emergency Department	6
(iv) Intensive Care Unit	2
(v) Dermatology and Allergy	2
(vi) Unknown	5
Diagnostics codes in Emergency Department	
(i) DT78.3 Quincke's edema	56.3%
(ii) DT78.4 Allergy unspecified	33.3%
(iii) DT88.6 Anaphylactic shock	2.0%
(iv) Miscellaneous	8.4%
Diagnostic codes in other departments	
DT78.3 Quincke's edema	59.6%
DT78.4 Allergy unspecified	15.9%
DT88.6 Anaphylactic shock	2.1%
Miscellaneous	18.1%
Unknown	4.3%

**Table 4:** Concomitant disease. Numbers do not add up to 100%, as some patients had more than one concomitant disease.

Disease	Frequency, *n*, *(%)*
Hypertension	97 *(92.4%)*
Diabetes	23 *(21.9%), *
Other ischemic heart disease	16 *(15.2%)*
Rheumatic disease	14 *(13.3%)*
Heart failure	13 *(12.4%)*
Hypercholesterolemia	13 *(12.4%)*
Allergic rhinitis	10 *(9.5%)*
Asthma	9 *(8.6%)*
COPD	6 *(5.7%)*
Atopic dermatitis	6 *(5.7%)*
Psychiatric disease	5 *(4.8%)*
Osteoporosis	4 *(3.8%)*
History of stroke	4 *(3.8%)*
Thyroid disease	3 *(2.9%)*
Cancer	3 *(2.9%)*

**Table 5:** Laboratory tests performed.

Laboratory tests	Result	% of cohort
Complement C1-inhibitor tests	Normal	72.4%
	Not tested	27.6%

HR test chronic urticaria	Negative	55.2%
	Not tested	40.0%
	Positive	2.9%
	Data missing	1.9%

Increased C-reactive protein	Range 7–126 (normal < 6 mg/L)	14.3%

Leukocytosis	More than 8.8 × 10^9^/L	15.2%

Tryptase	More than 12 ng/mL	5.7%

**Table 6:** Multivariate logistic regression analysis. Association between different factors and the need for admission. ^*∗*^Significant result, peripheral angioedema less likely to cause the patient to be admitted. ^1^High odds ratios for admission.

	Odds ratio	Standard error	*z*	*p* > *z*	95% confidence interval
Male versus female sex	0.74	0.40	−0.55	0.58	0.25–2.15
Urticaria	0.42	0.28	−1.33	0.19	0.11–1.52
Head and neck angioedema	**6.93** ^1^	9.05	1.48	0.14	0.53–98.49
Peripheral angioedema	0.14	0.14	−2.00	0.05^*∗*^	0.01–0.95
Smoking	**2.02** ^1^	1.29	1.10	0.27	0.57–7.06
Rash	1.05	0.58	0.10	0.92	0.35–3.11
Diabetes	1.78	1.14	0.91	0.37	0.50–6.23
Hypertension	0.72	0.68	−0.35	0.73	0.11–4.53
Ischemic heart disease	1.16	0.45	0.39	0.70	0.54–2.48
Heart failure	1.23	0.57	0.44	0.66	0.49–3.06
Atopic dermatitis	0.66	0.68	−0.40	0.69	0.08–5.01
Allergic rhinitis	**2.30** ^1^	2.15	0.89	0.37	0.37–14.39
Asthma	0.78	0.45	−0.43	0.67	0.25–2.42

## Additional Points

Institutions where the work was performed are as follows: collection of data: Department of Dermatology and Allergy Centre, Odense University Hospital, Sdr. Boulevard 29, Entrance 142, 5000 Odense C, Denmark, with associated physicians: Georg Authried, Sumangali Chandra Prasad, Kristine Appel U. Pallesen, Kawa Khaled Ajgeiy, Shailajah Kamaleswaran, and Anette Bygum; analysis and statistics: Department of Otorhinolaryngology, Head & Neck Surgery and Audiology, Rigshospitalet, University of Copenhagen, with associated physicians: Dr. Eva Rye Rasmussen and Dr. Christian von Buchwald.
