# Corrigendum to “Assessment of 105 Patients with Angiotensin Converting Enzyme-Inhibitor Induced Angioedema”

**DOI:** 10.1155/2018/9169414

**Published:** 2018-03-01

**Authors:** Eva Rye Rasmussen, Christian von Buchwald, Mia Wadelius, Sumangali Chandra Prasad, Shailajah Kamaleswaran, Kawa Khaled Ajgeiy, Georg Authried, Kristine Appel U. Pallesen, Anette Bygum

**Affiliations:** ^1^Department of Otorhinolaryngology, Head & Neck Surgery and Audiology, Rigshospitalet, University of Copenhagen, København, Denmark; ^2^Department of Medical Sciences, Clinical Pharmacology and Science for Life Laboratory, Uppsala University, Uppsala, Sweden; ^3^Department of Dermatology and Allergy Centre, Odense University Hospital, Odense, Denmark

In the article titled “Assessment of 105 Patients with Angiotensin Converting Enzyme-Inhibitor Induced Angioedema” [1], there was an error in the Results section where the text reading “In multivariate logistic regression analysis, angioedema located in the head and neck region was nonsignificantly associated with admission (odds ratio (OR) 5.9 (*p* = 0.15, 95% CI 0.53–98.49)), while angioedema in peripheral sites was significantly associated with not being admitted (OR 0.15 (*p* = 0.05 95% CI, 0.01–0.95) (Table 6))” should be corrected as follows.

“In multivariate logistic regression analysis, angioedema located in the head and neck region was nonsignificantly associated with admission (odds ratio (OR) 6.93 (*p* = 0.14, 95% CI 0.53–98.49)), while angioedema in peripheral sites was significantly associated with not being admitted (OR 0.15 (*p* = 0.05 95% CI, 0.01–0.95) (Table 6)).”
